# Melorheostosis: Clinical, radiological, and histopathological features with a literature review

**DOI:** 10.14814/phy2.70652

**Published:** 2025-11-10

**Authors:** Elif Koca, Şeyhmus Kavak, Bilal Demir, Nil Comunoglu, Fatih Kantarci, Oguzhan Koca

**Affiliations:** ^1^ Department of Internal Medicine, Cerrahpasa Faculty of Medicine Istanbul University‐Cerrahpasa Istanbul Turkey; ^2^ Department of Orthopaedics and Traumatology, Cerrahpasa Faculty of Medicine Istanbul University‐Cerrahpasa Istanbul Turkey; ^3^ Department of Radiology, Cerrahpasa Faculty of Medicine Istanbul University‐Cerrahpasa Istanbul Turkey; ^4^ Department of Pathology, Cerrahpasa Faculty of Medicine Istanbul University‐Cerrahpasa Istanbul Turkey; ^5^ Department of Immunology and Inflammation, Faculty of Medicine Imperial College London London UK

**Keywords:** bone remodeling, MAPK pathway, Melorheostosis, multimodal imaging, sclerosing bone dysplasia

## Abstract

Melorheostosis is a rare sclerosing bone dysplasia that can clinically and radiologically mimic common bone disorders, particularly in atypical presentations. Its heterogeneous manifestations and limited awareness among clinicians frequently contribute to diagnostic delays or misdiagnosis. We report the case of a 34‐year‐old woman with chronic forearm pain and a longstanding subcutaneous mass. She was initially misdiagnosed as having a metabolic bone disorder based on imaging and histopathology. However, subsequent evaluation with MRI and bone scintigraphy demonstrated eccentric cortical thickening and longitudinal sclerotic lesions involving the radius, olecranon, and first metacarpal. Normal biochemical markers and the absence of systemic involvement further supported a diagnosis of melorheostosis. Histopathological re‐examination confirmed lamellar cortical bone with osteoblastic rimming but remained nonspecific. This case underscores the importance of considering melorheostosis in the differential diagnosis of sclerosing bone disorders, especially when radiographic features are ambiguous. It highlights the critical role of multimodal imaging in establishing an accurate diagnosis, while histopathology provides complementary but not definitive evidence. Importantly, melorheostosis may also serve as a window into bone physiology, illustrating aberrant osteogenesis and dysregulated cortical remodeling. A better understanding of its molecular basis, particularly MAPK pathway alterations, may ultimately facilitate more targeted and effective treatments.

## 1 INTRODUCTION

Melorheostosis is a rare, benign mesenchymal bone dysplasia characterized by progressive cortical hyperostosis along the surfaces of existing bone. First described in 1922 by Léri and Joanny, the term derives from the Greek words *melos* (limb), *rhein* (flowing), and *ostosis* (bone formation), reflecting its distinctive radiological appearance often likened to “dripping candle wax” (Leri & Joanny, 1922). While the disease can affect any bone, it most commonly involves the appendicular skeleton, predominantly the unilateral long bones of the lower limbs (Wenokor et al., 2025).

Due to its rarity, comprehensive epidemiological data are limited. To date, approximately 400 cases have been reported in the literature, with no clear gender predilection (Iordache et al., 2023). Onset typically occurs in late adolescence or early adulthood (Kotwal & Clarke, 2017). Clinically, melorheostosis often follows an insidious and slowly progressive course. Pain is the most common symptom, but the spectrum ranges from mild discomfort to severe functional limitation depending on the degree of osseous and soft tissue involvement (Freyschmidt, 2001). Skeletal manifestations may be monostotic, polyostotic, monomelic, or hemimelic, with monomelic involvement being the most common presentation (Iordache et al., 2023). Cutaneous manifestations such as shininess, erythema, or scleroderma‐like changes, and soft tissue alterations including fibrosis, myosclerosis, and joint contractures, may also occur (Dong et al., 2024; Freyschmidt, 2001).

Diagnosis relies primarily on imaging. Radiographs typically demonstrate excessive cortical bone formation characterized by linear hyperostosis resembling “candle wax dripping” usually in the absence of laboratory abnormalities (Bhattacharyya, 2025). Computed tomography (CT) and magnetic resonance imaging (MRI) provide further characterization, whereas biopsy is reserved for ambiguous cases since no histological finding is pathognomonic (Freyschmidt, 2001). These radiological hallmarks reflect localized dysregulation of cortical remodeling, highlighting the relevance of melorheostosis as a clinical model of abnormal bone physiology.

The pathophysiology of melorheostosis remains incompletely understood. The historical sclerotomal hypothesis suggested that a postnatal segmental sensory nerve lesion was the underlying cause, leading to disease confined to a single sclerotome (Murray & McCredie, 1979). However, subsequent imaging‐based studies have challenged this view. In a whole‐body CT analysis of affected patients, a distribution corresponding to a single sclerotome was observed in only 17% of cases, indicating that the hypothesis cannot account for the majority of presentations (Jha et al., 2018).

Current evidence suggests that somatic activating mutations in bone tissue are the primary cause in most cases. Mutations in MAP2K1 and SMAD3 have been identified in classical and endosteal forms of the disease, while LEMD3 alterations have been reported in osteopoikilosis‐related cases (Bhattacharyya, 2025). These discoveries indicate that melorheostosis has a genetic basis and underscore its heterogeneity among sclerosing bone dysplasias.

Management remains symptomatic and multidisciplinary. Non‐steroidal anti‐inflammatory drugs (NSAIDs) and physiotherapy are first‐line, while bisphosphonates and denosumab have shown variable benefit in case reports (Byberg et al., 2018; Kumar et al., 2020; Sathish et al., 2021; Slimani et al., 2013). Surgical intervention may be indicated for patients with severe deformities, neurological complications, or persistent pain unresponsive to conservative measures.

Although its radiographic features can be distinctive, diagnostic challenges arise when melorheostosis mimics other sclerosing or metabolic bone disorders. Here, we present a diagnostically challenging case of melorheostosis with multimodal imaging and histopathological correlation, alongside a review of the literature, emphasizing its clinical significance and potential as a translational model of disordered bone remodeling.

## 2 CLINICAL AND TRANSLATIONAL SIGNIFICANCE: A CASE OF MELORHEOSTOSIS

A 34‐year‐old woman with no significant past medical history presented with persistent pain in her left forearm. She denied any trauma. On physical examination, a firm, non‐tender, immobile subcutaneous mass measuring approximately 2 cm was palpable on the ventral aspect of the left distal forearm. No overlying skin changes were observed. The patient reported a 15‐year history of this lesion with gradual enlargement. Family history was unremarkable.

**FIGURE 1:**
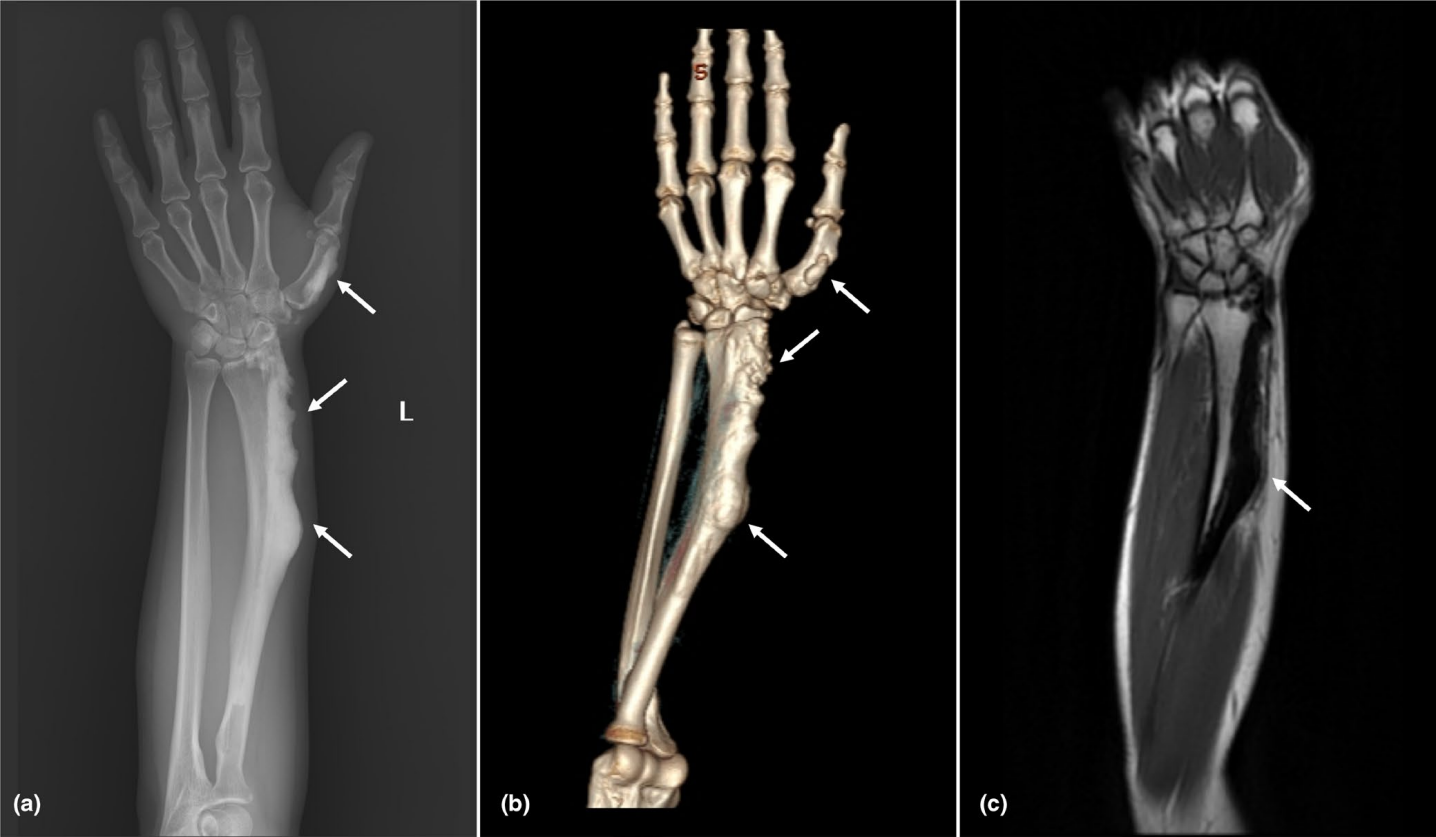
Multimodal imaging of melorheostotic lesions involving the left radius and first metacarpal. (a) Plain radiograph showing irregular, eccentric cortical thickening along the lateral aspect of the left radius and first metacarpal bone, with intramedullary extension. White arrows indicate the sites of cortical involvement. (b) Three‐dimensional reconstruction of the forearm and hand, demonstrating an eccentrically located extraosseous lesion arising from the cortex. White arrows highlight the abnormal cortical thickening. (c) Coronal T1‐weighted MRI showing a well‐circumscribed, eccentrically located lesion involving the distal two‐thirds of the left radius. The lesion appears hypointense and lacks soft tissue extension. The white arrow indicates the site of cortical involvement.

Six months earlier, she had been evaluated at another facility, where plain radiography revealed a bony mass at the distal radius (Figure 1). MRI demonstrated nodular cortical thickening along the dorsal and lateral aspects of the radius, extending from the proximal diaphysis to the distal metaphysis, with a maximum cortical thickness of 17 mm. Biopsy at that time showed compact sclerotic bone with osteoclastic resorption, new bone formation, and areas of necrotic lamellae. Although no distinct lesion was identified, the findings raised suspicion for a metabolic bone disorder.

Upon referral to our center, whole‐body bone scintigraphy demonstrated increased uptake confined to the known lesion, with a linear distribution on delayed images and no evidence of increased vascularity (Figure S1). These findings favored a benign sclerosing bone pathology. Laboratory results were within normal limits: alkaline phosphatase (ALP) at 51 U/L, bone‐specific ALP at 11.6 U/L, parathyroid hormone at 41 pg/mL, calcium at 9 mg/dL, and phosphorus at 3 mg/dL. However, 25‐hydroxyvitamin D was notably low at 4 ng/mL (Table 1).

**TABLE 1:** Patient's laboratory findings.

Parameter	Result	Reference range
Leukocyte count	7800/μL	4500–10,000
Neutrophil count	4700/μL	2000–6000
Hemoglobin	14.1 g/dL	12–16
CRP	0.7 mg/L	0–5
ESR	13 mm/h	0–20
Creatinine	0.6 mg/dL	0.5–0.9
Urea	17 mg/dL	17–49
AST	11 IU/L	<32
ALT	12 IU/L	<33
LDH	125 U/L	<250
Na	140 mEq/L	136–145
Cl	103 mEq/L	98–107
K	4.6 mEq/L	3.5–5.1
Ca	9 mg/dL	8.4–10.2
iCa	4.4 mg/dL	4.3–5.1
P	3 mg/dL	2.5–4.5
Mg	2 mg/dL	1.6–2.6
Total protein	7 g/dL	6.4–8.3
Albumin	4.5 g/dL	3.5–5
ALP	51 U/L	35–105
Bone‐specific ALP	11.6 U/L	7.7–21.3
Parathyroid hormone	41 pg/mL	15–65
25‐OH Vitamin D	4 ng/mL	>30

Abbreviations: ALP, alkaline phosphatase; ALT, alanine aminotransferase; AST, aspartate aminotransferase; CRP, C‐reactive protein; ESR, erythrocyte sedimentation rate; iCa, ionized calcium; LDH, lactate dehydrogenase.

**FIGURE 2:**
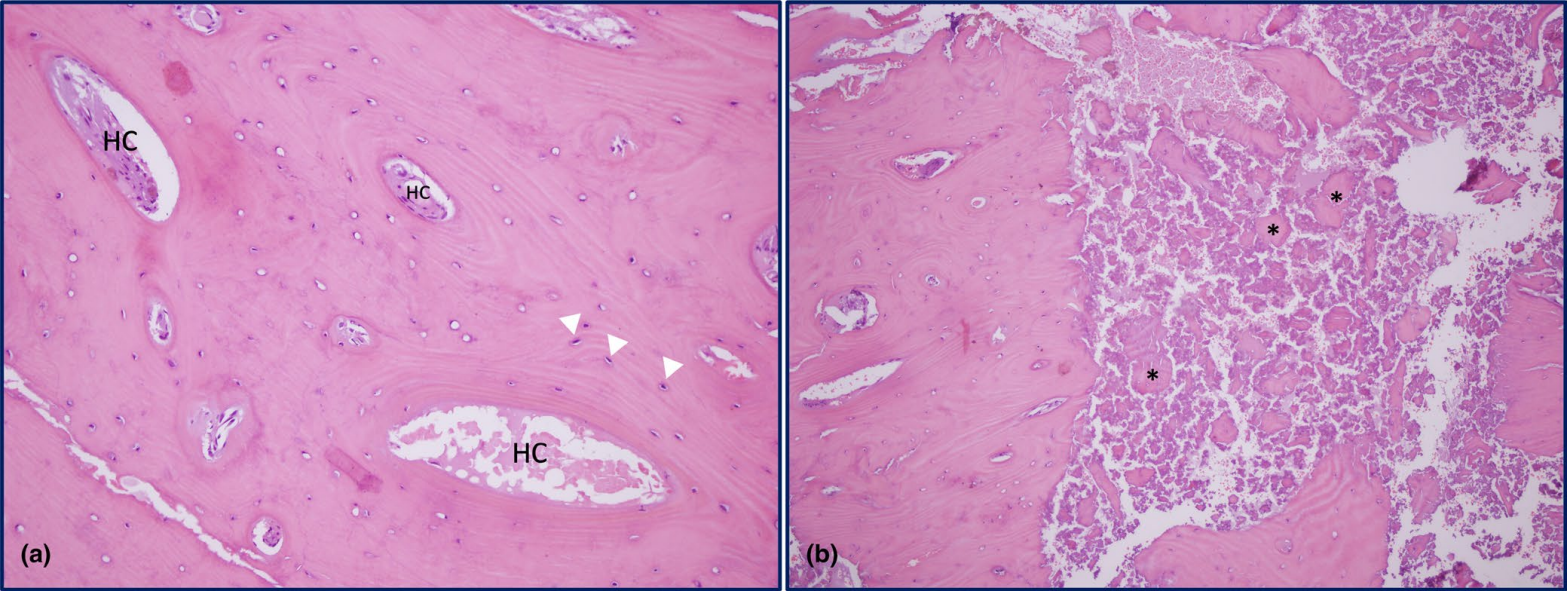
Histopathological features of the radial cortical lesion (H&E, ×200). (a) Lamellar cortical bone with increased density and a markedly elevated number of Haversian systems, consistent with compact sclerotic remodelling. Haversian canals (HC) are labelled, and osteocytes are marked with white arrowheads. (b) Adjacent area demonstrating newly deposited, unmineralized osteoid matrix with irregular trabecular architecture and increased cellularity. Asterisks highlight osteoid‐rich regions.

Given the patient's young age, long‐standing history, and normal ALP levels, the initial diagnosis was reconsidered. Repeat MRI confirmed a well‐circumscribed, eccentric cortical sclerotic lesion, hypointense on both T1‐ and T2‐weighted sequences, extending from the proximal diaphysis to the distal metaphysis of the radius and involving the radial styloid (Figure 1). Additional lesions of similar morphology were present at the olecranon and the first metacarpal (Figure S2). These imaging features were highly characteristic of melorheostosis. Histopathological re‐examination of the biopsy specimen revealed lamellar cortical bone with dense sclerosis and prominent osteoblastic rimming, further supporting the diagnosis (Figure 2). In addition, some osteocyte lacunae appeared empty, suggesting osteocyte loss, and spindle‐shaped osteoprogenitor or bone marrow stromal‐like cells were observed along the Haversian canals, consistent with focal bone remodeling.

The patient was referred to the orthopedic team. Given the lesion's stability and absence of neurological compromise, conservative management with NSAIDs was advised. During 1 year of follow‐up, she remained clinically stable under NSAID therapy, with no new symptoms or progression of complaints.

## 3 DISCUSSION

Melorheostosis is a rare sclerotic bone dysplasia that remains diagnostically challenging owing to its rarity, wide clinical spectrum, and heterogeneous imaging features. Although the “dripping candle wax” appearance is considered classic, five radiological patterns have been described: osteoma‐like, classic candle wax, myositis ossificans‐like, osteopathia striata‐like, and mixed forms (Freyschmidt, 2001). Among these, the osteoma‐like form may be more frequent than the classic pattern, but its lack of pathognomonic features often results in misinterpretation, mimicking entities such as osteoma or parosteal osteosarcoma (Freyschmidt, 2001; Han et al., 2022; Jha et al., 2018; Smith et al., 2017). Recognizing these radiological variants is critical to avoid diagnostic delay and unnecessary invasive interventions.

Accurate diagnosis of melorheostosis requires integration of clinical, radiological, and, when indicated, histopathological findings, as laboratory results are typically unremarkable. Although rare cases with elevated ALP have been reported in polyostotic disease (Donáth et al., 2002), most patients demonstrate normal biochemical profiles. While plain radiographs are the initial imaging modality, CT and MRI are often essential for a comprehensive assessment of bone involvement, soft tissue changes, or neurovascular compression. CT can offer superior delineation of cortical thickening and lesion extent, whereas MRI is particularly useful for detecting bone marrow oedema, periosteal involvement, and soft tissue changes (Hurley‐Novatny et al., 2021; Judkiewicz et al., 2001). Melorheostotic lesions typically appear hypointense on both T1‐ and T2‐weighted sequences, reflecting dense sclerosis, while associated soft tissue components may show variable enhancement (Judkiewicz et al., 2001).

Nuclear medicine techniques provide complementary information. Technetium‐99 m scintigraphy typically demonstrates moderate delayed‐phase tracer uptake correlating with increased osteoblastic activity and local bone turnover (Davis et al., 1992; Iordache et al., 2023). This distinguishes melorheostosis from osteopoikilosis and osteopathia striata, which generally show no increased uptake. Additionally, 18F‐NaF PET/CT has been reported as a complementary modality for evaluating disease activity and performing whole‐body screening (Papadakis et al., 2017).

Histopathological examination usually demonstrates dense cortical bone, woven bone, hypervascularity, unmineralized osteoid, and increased porosity with abundant Haversian systems (Fick et al., 2019). Although non‐specific, biopsy remains valuable to exclude malignancies such as osteosarcoma and malignant fibrous histiocytoma (Baer et al., 1994; Bostman et al., 1987). Importantly, histopathology aligns with underlying disturbances in bone remodeling, reinforcing the role of melorheostosis as a localized disorder of osteogenesis.

Cutaneous manifestations may provide additional diagnostic clues. Hyperpigmentation, erythematous macules, or scleroderma‐like plaques frequently overlie affected bone (Smith et al., 2017). The presence of nevi or dermatofibrosis should raise suspicion for Buschke‐Ollendorff syndrome, characterized by skin lesions associated with osteopoikilosis (Mumm et al., 2007). Notably, skin biopsies from affected areas have been shown to harbor the same somatic MAP2K1 mutations found in underlying bone, suggesting that skin sampling may serve as a less invasive diagnostic adjunct in selected patients (Jha et al., 2021).

There is no established consensus on optimal management of melorheostosis, and treatment is largely symptom‐directed. Conservative treatment with NSAIDs and physiotherapy is preferred for asymptomatic or mildly symptomatic patients. Additionally, bisphosphonates and denosumab have shown variable symptomatic benefit in case series (Byberg et al., 2018; Kumar et al., 2020; Sathish et al., 2021; Slimani et al., 2013). Interestingly, the mechanism of bisphosphonates may extend beyond antiresorptive effects, encompassing anti‐angiogenic and immunomodulatory actions that may explain their analgesic properties in melorheostosis (Wood et al., 2002). However, case series investigating bisphosphonate use in melorheostosis report only short‐term follow‐up data, limiting our understanding of their long‐term efficacy (Byberg et al., 2018; Kumar et al., 2020; Sathish et al., 2021; Slimani et al., 2013). Denosumab, through RANKL inhibition, targets osteoclast‐mediated resorption, although clinical responses have been inconsistent. Surgical intervention is reserved for patients with severe deformities, neurovascular compromise, or refractory pain, but recurrence rates are high, often necessitating repeat procedures (Slimani et al., 2013). These limitations underscore the need for molecularly informed therapies.

## 4 PHYSIOLOGY AND TRANSLATIONAL SIGNIFICANCE: LESSONS FROM A RARE BONE DYSPLASIA

Although rare, melorheostosis has recently emerged as a valuable model for exploring bone physiology through advances in molecular genetics. Early evidence from familial cases of osteopoikilosis and Buschke–Ollendorff syndrome demonstrated that germline LEMD3 loss‐of‐function mutations disrupt BMP/TGF‐β antagonism, occasionally in association with melorheostosis, thus providing the first link between dysregulated TGF‐β signaling and sclerosing bone dysplasias (Hellemans et al., 2004).

Decades later, high‐resolution sequencing of sporadic melorheostosis revealed recurrent somatic activating mutations in MAP2K1 confined to affected bone (Kang et al., 2018). These variants, localized to the negative regulatory domain of MEK1, cause constitutive MAPK/ERK pathway activation, increased osteoblast proliferation, and impaired mineralization, thereby explaining the exuberant periosteal new bone formation characteristic of classical melorheostosis. Independent multi‐omics analyses have expanded this mutational spectrum to include catalytic domain variants, while also identifying cases without detectable mutations, underscoring genetic heterogeneity and the likelihood of additional undiscovered loci (De Ridder et al., 2020).

A distinct subset of patients with endosteal lesions has been shown to carry somatic activating mutations in SMAD3, resulting in constitutive TGF‐β/SMAD signaling. In contrast to MAP2K1‐mutant lesions, which demonstrate abundant osteoid and impaired mineralization, SMAD3 lesions exhibit denser cortical bone, enhanced mineralization, and increased osteoblast differentiation, defining a genotype–phenotype correlation that accounts for divergent radiographic and histological subtypes (Kang et al., 2020). Together, these studies firmly establish melorheostosis as a genetically heterogeneous disorder driven by perturbations in both MAPK and TGF‐β/SMAD cascades, two central regulators of osteoblast biology and cortical bone remodeling.

More recently, comprehensive reviews have integrated these findings, recognizing KRAS as an additional, though less frequent, somatic driver and highlighting the role of angiogenic signaling, particularly VEGF secretion by MAP2K1‐mutant fibroblasts and osteoblasts, in driving abnormal bone formation (Bhattacharyya, 2025). These insights not only delineate the mutational spectrum of melorheostosis but also extend its relevance to broader areas of skeletal biology and translational therapeutics.

An additional layer of complexity arises from somatic mosaicism: identical MAP2K1 variants have been detected in both bone and overlying skin, supporting a developmental model in which post‐zygotic mutations during embryogenesis seed localized skeletal regions (Kang et al., 2018). This observation has direct translational implications, suggesting that accessible tissues such as skin may serve as surrogates for invasive bone biopsy in establishing diagnosis and studying pathogenesis.

From a physiological perspective, melorheostosis represents a “natural experiment” in localized disordered bone formation. It demonstrates how focal mutations confined to a subset of cells can disrupt the equilibrium of bone turnover, leading to excessive cortical sclerosis despite otherwise normal systemic bone metabolism. Such insights extend beyond this rare dysplasia: they illustrate how dysregulation of MAPK and TGF‐β pathways, whether through germline mutations, somatic mosaicism, or acquired alterations, may profoundly influence osteoblast function, bone remodeling, and tissue microarchitecture.

Therefore, melorheostosis holds translational relevance well beyond its diagnostic and therapeutic challenges. By linking rare clinical observations with fundamental mechanisms of osteoblast signaling, angiogenesis, and cortical remodeling, it provides a framework for pathway‐specific therapies. Lessons derived from this condition may ultimately inform not only the management of melorheostosis but also the development of targeted interventions for more common skeletal disorders in which MAPK and TGF‐β signaling play critical roles, including osteoporosis and metabolic sclerosing dysplasias.

## 5 CONCLUSION

Melorheostosis is a rare sclerotic bone dysplasia with heterogeneous clinical features and variable imaging findings that often complicate diagnosis and management. Recent genetic discoveries, particularly somatic mutations in MAP2K1 and SMAD3 and occasional LEMD3 alterations, implicate dysregulation of MAPK and TGF‐β/SMAD signaling pathways. These advances establish melorheostosis not only as a diagnostic challenge but also as a unique model of localized disordered bone remodeling. Future research should focus on clarifying its molecular mechanisms and developing targeted, consensus‐driven treatment strategies.

## AUTHOR CONTRIBUTIONS

EK conceptualized the case report, assisted with data collection, contributed to the literature review, drafted the manuscript, and ensured the accuracy of references. OK conducted the initial patient assessment, provided clinical care, contributed to the case presentation and discussion, revised the manuscript for important intellectual content, and obtained informed consent from the patient. ŞK, BD, FK, and NC contributed to the case presentation and discussion. All authors critically revised the manuscript for important intellectual content and approved the final version. All authors agree to be accountable for all aspects of the work and to ensure that any questions related to the accuracy or integrity of any part of the work are appropriately investigated and resolved.

## FUNDING INFORMATION

This research did not receive any specific grant from funding agencies in the public, commercial, or not‐for‐profit sectors.

## CONFLICT OF INTEREST STATEMENT

The authors have no conflicts of interest to declare.

## ETHICS STATEMENT

This study was conducted at Cerrahpasa Faculty of Medicine, Istanbul University‐Cerrahpasa, in accordance with the ethical standards of the World Medical Association Declaration of Helsinki (2013). Institutional Review Board (IRB) approval was not required for this single‐patient case report. Written informed consent for participation and for publication of anonymised clinical and imaging data was obtained from the patient.

## Supporting information


**Figure S1.** Whole‐body bone scintigraphy using (99 m) Tc‐methylene diphosphonate (MDP). (a) Whole‐body planar scintigraphy obtained 3 h after tracer injection demonstrates linear increased uptake localized to the distal radius of the left forearm (red oval), while no abnormal uptake is observed in the contralateral right forearm. (b) Regional scintigraphy of the forearms shows focal, linear increased tracer uptake at the distal left radius (dashed outline), corresponding to the site of cortical sclerosis identified on radiographs and MRI.
**Figure S2.** Cross‐sectional imaging of the melorheostotic lesion in the first metacarpal. (a) Coronal T1‐weighted MRI demonstrates a well‐circumscribed, hypointense cortical lesion involving the lateral aspect of the first metacarpal (red arrow). (b) Coronal CT shows cortical thickening and sclerosis of the corresponding region (red arrow), consistent with melorheostotic involvement.

**File S1.** Completed CARE checklist.

## Data Availability

Data will be made available on request.
