# Supplementary figures and images for: Melorheostosis: Clinical, radiological, and histopathological features with a literature review
Source: Physiol Rep. 2025 Nov 10;13(21):e70652. doi: 10.14814/phy2.70652 (PMC12602463; doi:10.14814/phy2.70652)

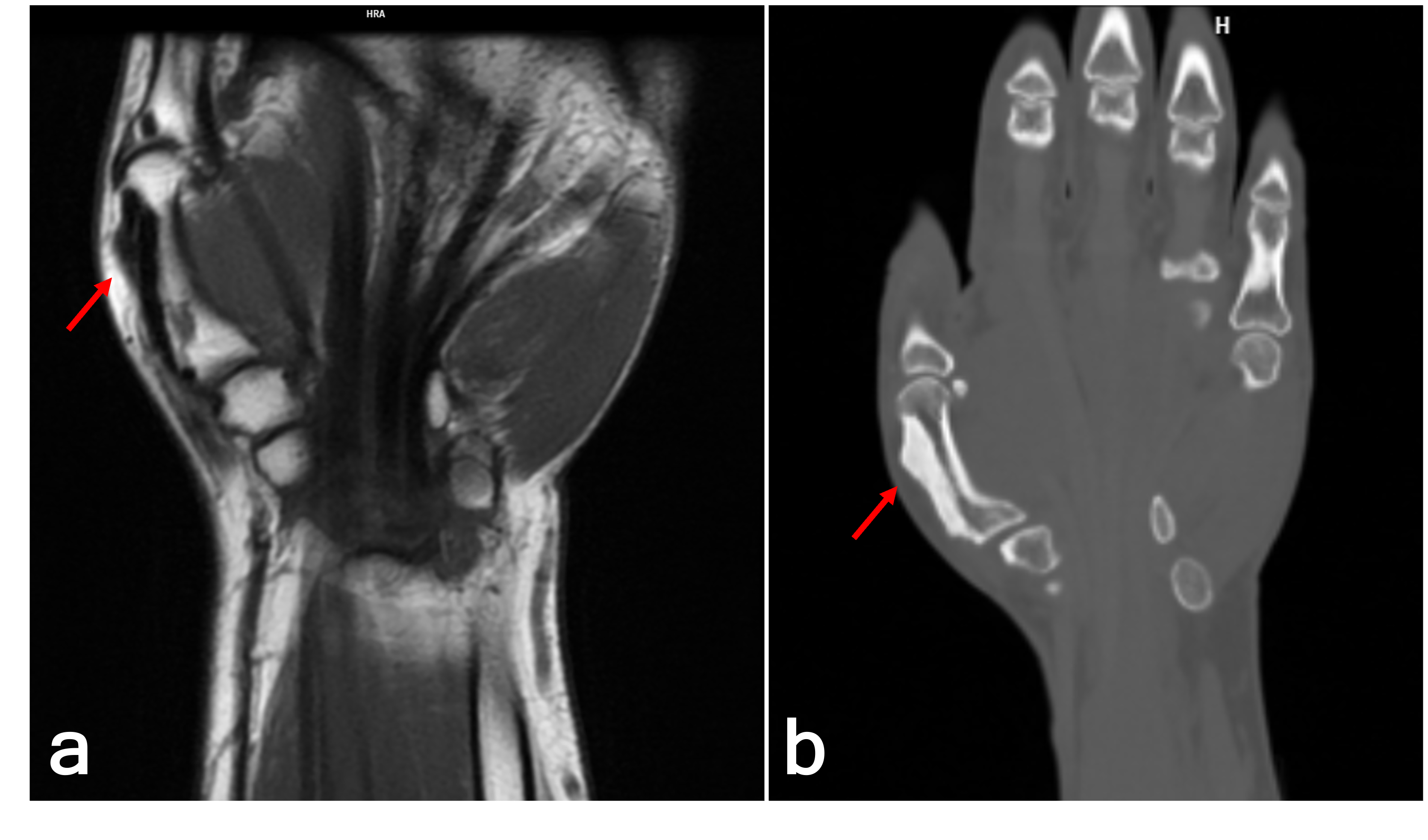

Supplement: Supplementary file 1 — Figure S1. Whole‐body bone scintigraphy using (99 m) Tc‐methylene diphosphonate (MDP). (a) Whole‐body planar scintigraphy obtained 3 h after tracer injection demonstrates linear increased uptake localized to the distal radius of the left forearm (red oval), while no abnormal uptake is observed in the contralateral right forearm. (b) Regional scintigraphy of the forearms shows focal, linear increased tracer uptake at the distal left radius (dashed outline), corresponding to the site of cortical sclerosis identified on radiographs and MRI. Figure S2. Cross‐sectional imaging of the melorheostotic lesion in the first metacarpal. (a) Coronal T1‐weighted MRI demonstrates a well‐circumscribed, hypointense cortical lesion involving the lateral aspect of the first metacarpal (red arrow). (b) Coronal CT shows cortical thickening and sclerosis of the corresponding region (red arrow), consistent with melorheostotic involvement. [file PHY2-13-e70652-s001.zip › PHYSREP-2025-09-835-s02.png]

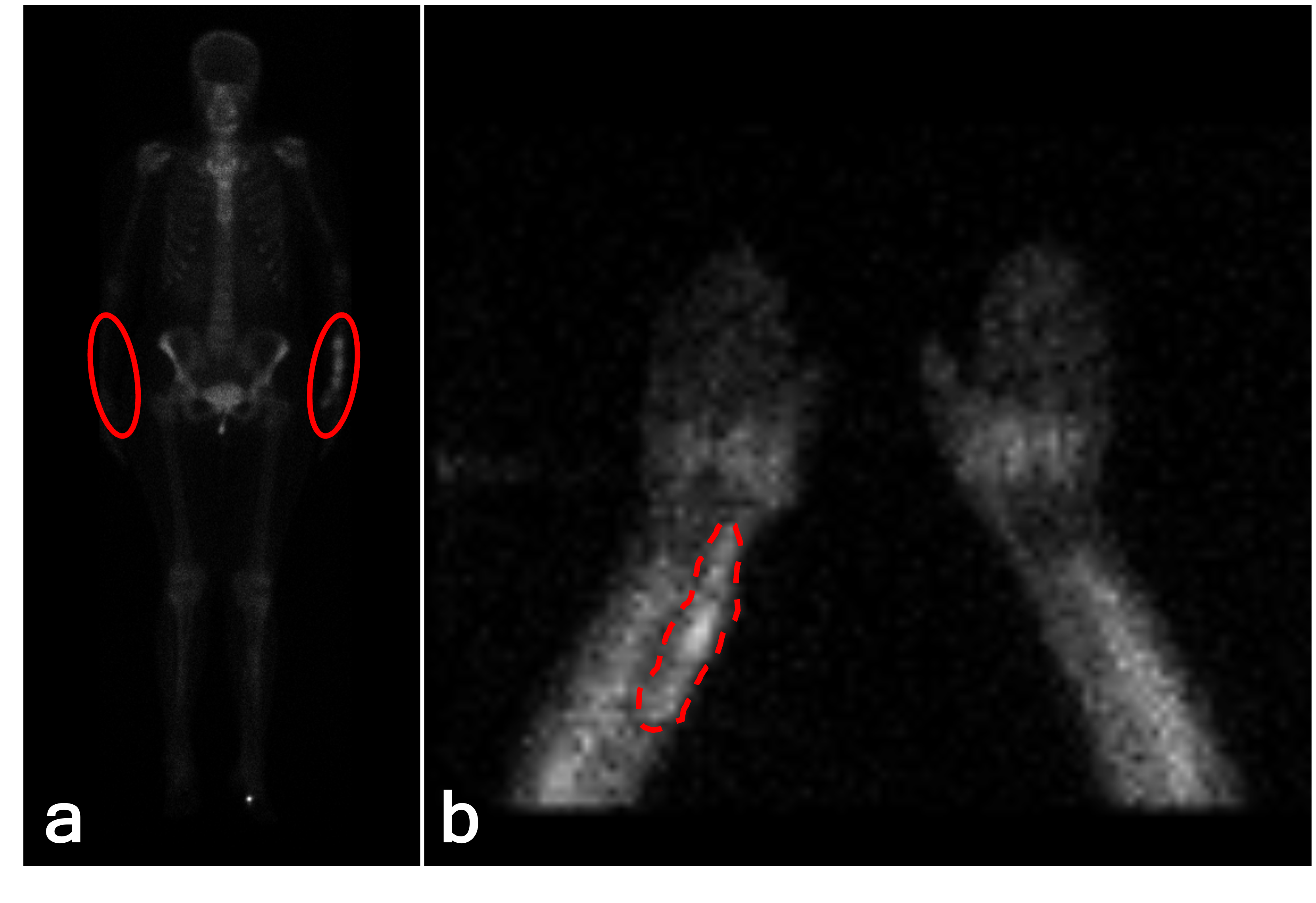

Supplement: Supplementary file 1 — Figure S1. Whole‐body bone scintigraphy using (99 m) Tc‐methylene diphosphonate (MDP). (a) Whole‐body planar scintigraphy obtained 3 h after tracer injection demonstrates linear increased uptake localized to the distal radius of the left forearm (red oval), while no abnormal uptake is observed in the contralateral right forearm. (b) Regional scintigraphy of the forearms shows focal, linear increased tracer uptake at the distal left radius (dashed outline), corresponding to the site of cortical sclerosis identified on radiographs and MRI. Figure S2. Cross‐sectional imaging of the melorheostotic lesion in the first metacarpal. (a) Coronal T1‐weighted MRI demonstrates a well‐circumscribed, hypointense cortical lesion involving the lateral aspect of the first metacarpal (red arrow). (b) Coronal CT shows cortical thickening and sclerosis of the corresponding region (red arrow), consistent with melorheostotic involvement. [file PHY2-13-e70652-s001.zip › PHYSREP-2025-09-835-s01.png]
