# Supplementary material for: Melorheostosis: Clinical, radiological, and histopathological features with a literature review
Source: Physiol Rep. 2025 Nov 10;13(21):e70652. doi: 10.14814/phy2.70652 (PMC12602463; doi:10.14814/phy2.70652)
Supplement: Supplementary file 2 — File S1. Completed CARE checklist. [file PHY2-13-e70652-s002.pdf]

CARE
case report guidelines

CARE Checklist of information to include when writing a case report

(cc) BY-NC-ND

✓

Topic	Item	Checklist item description	Reported on Line
**Title**	**1**	The diagnosis or intervention of primary focus followed by the words "case report"	1
**Key Words**	**2**	2 to 5 key words that identify diagnoses or interventions in this case report, including "case report"	43-44
**Abstract (no references)**	**3a**	Introduction: What is unique about this case and what does it add to the scientific literature?	23-26
	**3b**	Main symptoms and/or important clinical findings	27-31
	**3c**	The main diagnoses, therapeutic interventions, and outcomes	31-34
	**3d**	Conclusion—What is the main "take-away" lesson(s) from this case?	35-42
**Introduction**	**4**	One or two paragraphs summarizing why this case is unique (**may include references**)	46-90
**Patient Information**	**5a**	De-identified patient specific information.	92-93
	**5b**	Primary concerns and symptoms of the patient.	93-97
	**5c**	Medical, family, and psycho-social history including relevant genetic information	96-97
	**5d**	Relevant past interventions with outcomes	98-104
**Clinical Findings**	**6**	Describe significant physical examination (PE) and important clinical findings.	92-110
**Timeline**	**7**	Historical and current information from this episode of care organized as a timeline	92-138
**Diagnostic Assessment**	**8a**	Diagnostic testing (such as PE, laboratory testing, imaging, surveys).	105-129
	**8b**	Diagnostic challenges (such as access to testing, financial, or cultural)	105-129
	**8c**	Diagnosis (including other diagnoses considered)	105-129
	**8d**	Prognosis (such as staging in oncology) where applicable	137-140
**Therapeutic Intervention**	**9a**	Types of therapeutic intervention (such as pharmacologic, surgical, preventive, self-care)	137-140
	**9b**	Administration of therapeutic intervention (such as dosage, strength, duration)	137-140
	**9c**	Changes in therapeutic intervention (with rationale)	
**Follow-up and Outcomes**	**10a**	Clinician and patient-assessed outcomes (if available)	137-140
	**10b**	Important follow-up diagnostic and other test results	
	**10c**	Intervention adherence and tolerability (How was this assessed?)	
	**10d**	Adverse and unanticipated events	
**Discussion**	**11a**	A scientific discussion of the strengths AND limitations associated with this case report	143-245
	**11b**	Discussion of the relevant medical literature **with references**.	143-245
	**11c**	The scientific rationale for any conclusions (including assessment of possible causes)	143-245
	**11d**	The primary "take-away" lessons of this case report (without references) in a one paragraph conclusion	246-253
**Patient Perspective**	**12**	The patient should share their perspective in one to two paragraphs on the treatment(s) they received	
**Informed Consent**	**13**	Did the patient give informed consent? Please provide if requested	**Yes** ☑ **No** ☐
